# Altered Peripheral Blood Mononuclear Cell (PBMC) Mitochondrial Coupling and Complex IV Respiration in Severe Aortic Stenosis Before and After Transcatheter Aortic Valve Replacement (TAVR)

**DOI:** 10.3390/jcdd13100480

**Published:** 2026-09-23

**Authors:** Benjamin Marchandot, Anne-Laure Charles, Kerem Demir, Cedric Momas, Samy Talha, Michel Kindo, Patrick Ohlmann, Margherita Giannini, Alain Meyer, Olivier Morel, Bernard Geny

**Affiliations:** 1 Cardiology Department, Strasbourg University Hospital, 1 Place de L’Hôpital, 67000 Strasbourg, France; 2 UR 3072, “Mitochondria, Oxidative Stress and Muscle Plasticity”, Biomedicine Research Center of Strasbourg (CRBS), Faculty of Medicine, University of Strasbourg, 67000 Strasbourg, France; 3 Department of Physiology and Functional Explorations, Strasbourg University Hospital, 67000 Strasbourg, France; 4 Cardiac Surgery Department, Strasbourg University Hospital, 1 Place de L’Hôpital, 67000 Strasbourg, France; 5 FMTS, Biomedicine Research Center of Strasbourg, UR 3074, Translational Cardiovascular Medicine, University of Strasbourg, 1 Rue Eugène Boeckel, 67000 Strasbourg, France

**Keywords:** aortic stenosis (AS), transcatheter aortic valve replacement (TAVR), peripheral blood mononuclear cells (PBMCs), mitochondria, mitochondrial respiration and coupling

## Abstract

Background: Circulating peripheral blood mononuclear cell (PBMC) mitochondrial respiration is impaired in heart failure and transplanted patients. Severe aortic stenosis (AS), characterized by pressure overload leading to cardiac mitochondrial dysfunction, might also be associated with PBMC mitochondrial respiration alterations, likely related to systemic inflammation and left heart hypertrophy and diastolic dysfunction. Population and Methods: We determined PBMC mitochondrial respiration by high-resolution respirometry (Oroboros Instruments) in 9 healthy subjects and 25 AS patients before and until 30 days after transcatheter aortic valve replacement (TAVR) and investigated potential correlates with the clinical, biological and echocardiographic characteristics of the patients. Results: AS patients with a transaortic valvular gradient of 45 mm Hg were older and demonstrated slightly impaired renal function, inflammation markers, increased brain natriuretic peptide, left ventricular hypertrophy, and impaired diastolic function. PBMC mitochondrial coupling was decreased in the AS patients compared to controls (2.12 ± 0.10 vs. 2.76 ± 0.17, *p* = 0.003) and mitochondrial complex IV respiration was increased (31.07 ± 2.84 vs. 18.69 ± 2.09, *p* = 0.017). TAVR did not modify the alterations. Interestingly, increased complex IV mitochondrial respiration was associated with reduced mortality (*p* < 0.01). Conclusions: Severe aortic stenosis is associated with bioenergetic remodeling of circulating mononuclear cells that were not significantly associated with valvular hemodynamic impairment and ventricular function, before and after the mechanical relief of afterload. Nevertheless, future studies will be useful to determine whether such systemic immuno-metabolic phenotype, particularly PBMC mitochondrial complex IV respiration stimulation, may contribute to reduce the risk following TAVR.

## 1. Introduction

Degenerative aortic stenosis is a progressive valvular disease with a prevalence exceeding 1% among valvular heart abnormalities in the general population [1]. Traditionally, it was regarded as passive, age-dependent wear-and-tear process resulting from progressive valvular degeneration [2]. However, accumulating evidence has redefined aortic stenosis as an active immunogenic disease characterized by complex inflammatory and immune-mediated mechanisms that drive its initiation and progression [3]. Calcific Aortic Stenosis (AS), the most common and clinically significant form of aortic stenosis, is an active inflammatory and osteogenic disease characterized by progressive valvular calcification rather than passive degeneration. Initiated by chronic mechanical stress and lipid deposition. AS-related chronic pressure overload imposes left ventricular hypertrophy and substantial metabolic stress on the myocardium, causing alteration of substrate utilization and impaired myocardial energetics [4], including mitochondria dysfunction [5], together with cardiac remodeling and possibly decompensation. Similar alterations have been observed in the right ventricle when responding to pressure overload after congenital heart disease [6].

At the valvular level, calcified stenotic aortic valves exhibit increased reactive oxygen species production [7,8]; recently, human aortic valve interstitial cells isolated from patients with AS were shown to display impaired mitochondrial respiration, accompanied by reduced ATP production and coupling efficiency [9].

However, evaluating AS by investigating myocardial mitochondrial function directly in patients poses severe clinical challenges. Endomyocardial biopsy is an invasive procedure requiring high technical expertise, and routine endomyocardial biopsies are generally restricted to the right heart, failing to accurately reflect the pathology of the left ventricle, which bears the direct pressure overload of AS. Despite the expertise, cardiac biopsy still carries prohibitive procedural risks [10,11]. Nowadays, many severe AS patients do not undergo surgical procedures because of significant risks [12] and transcatheter aortic valve replacement (TAVR) represents a rapidly developing, minimally invasive field for treating severe AS or even aortic insufficiency during long-term left ventricular assist device support, likely offering superior therapeutic outcomes in older and frail patients compared to conventional surgery [13,14,15].

Interestingly, the screening of 40,000 patients suffering from AS showed that patients classified with symptomatic moderate AS share a nearly identical survival and hospitalization profile to asymptomatic severe patients [16].

This suggests the need for other biomarkers of AS. In this view, peripheral blood mononuclear cells (PBMCs) might be interesting since impaired mitochondrial respiration was observed in several pathologies, including in COVID-19 patients and in patients presenting with cardiovascular diseases [17,18]. In heart failure, impaired PBMC mitochondrial respiration has been shown to reflect disease severity and to be linked to inflammation, oxidative stress, and adverse cardiac remodeling. These findings suggest that circulating PBMCs are not merely passive biomarkers but may represent systemic manifestations of ongoing cardiac pathology [19,20,21]. Furthermore, during heart transplantation, minor changes in cardiac diastolic function were reflected in PBMC mitochondrial respiration. This observation further supports the sensitivity of PBMCs to cardiac dysfunction, extending their potential utility beyond advanced heart failure [22].

Since aortic stenosis shares several pathophysiological mechanisms with heart failure, including chronic inflammation and progressive cardiac remodeling and left heart diastolic and/or systolic dysfunctions, we hypothesized that impaired PBMC mitochondrial respiration might be associated with AS severity and correspond to systemic factors and cardiac remodeling. Further, TAVR quickly reduced the reactive oxygen species superoxide anion [23]. This occurred together with immediate reduction in the trans-aortic gradient and therefore pressure overload. As such, we investigated whether potential abnormalities in PBMC mitochondrial respiration might be reversed three and thirty days after TAVR.

We therefore determined PBMC mitochondrial respiratory parameters in patients with severe aortic stenosis compared to healthy controls and investigated whether systemic or cardiac characteristics might be linked to AS severity. We thereby evaluated the potential of PBMC bioenergetics as a novel, non-invasive biomarker in aortic stenosis and investigated possible normalization after successful TAVR.

## 2. Population and Methods

### 2.1. Population and Study Design

Twenty-five patients with severe aortic stenosis needing TAVR were enrolled and compared to nine healthy controls, considering clinical characteristics and investigations of PBMCs. For TAVR, the usual clinical procedure was performed and commercially available valves, such as the balloon expandable Edwards SAPIEN XT or S3 prosthesis (Edwards Life sciences LLC, Irvine, CA, USA) and the self-expandable CoreValve or Evolute-R (Medtronic CV, Irvine, CA, USA), were used. The data regarding AS patients were collected by systematic medical chart review.

The study was conducted in accordance with the Declaration of Helsinki and approved by the Ethics Committee of the Faculties of Medicine, Dentistry, Pharmacy, Nursing Schools, Physiotherapy, Midwifery and Hospitals of Strasbourg (CE-2016-81) and written informed consent was obtained from all participants.

For PBMC analysis, 30 mL blood samples were obtained for mitochondrial respiration measurement through femoral vein access 10 min prior to valve implantation. Antecubital vein puncture was additionally performed at 3 and 30 days follow up. The blood samples were collected in lithium heparin tubes (BD Vacutainer Brand, Dickinson and Company, Franklin Lakes, NJ, USA) and processed one hour after withdrawal. Usual clinical, biological and echocardiographic data using conventional well-controlled methods were obtained in the patients with aortic stenosis before the TAVR procedure.

### 2.2. PBMC Isolation and Mitochondrial Assessments

#### 2.2.1. Extraction of Circulating Peripheral Blood Mononuclear Cells (PBMCs)

Briefly, whole blood was carefully layered onto a Ficoll density gradient (Lymphocyte Separation Medium, Eurobio, Courtaboeuf, France) and centrifuged at 2100 rpm for 25 min at 18 °C without brake. The PBMC layer was collected, washed with Dulbecco’s Phosphate-Buffered Saline (DPBS; HyClone, South Logan, UT, USA), and centrifuged at 1600 rpm for 10 min at 18 °C. When residual erythrocytes were present, the cell suspension was incubated with a Versalyse-type solution for 20 min to lyse red blood cells without affecting leukocytes, followed by an additional DPBS wash. Finally, isolated PBMCs were quantified using a Muse Cell Analyzer flow cytometer (Merck Millipore, Darmstadt, Germany).

#### 2.2.2. Mitochondrial Coupling Determination

Mitochondrial coupling is inferred from the respiratory control ratio (RCR) (OXPHOS CI/CI leak), which indicates the degree of coupling between oxidation and phosphorylation. OXPHOS CI and CI leak represent the maximal and the basal mitochondrial respiratory rates, respectively.

#### 2.2.3. Mitochondrial Respiration of PBMCs

Oxygen consumption was measured in permeabilized peripheral blood mononuclear cells (PBMCs, 2.5 × 10^6^ cells/mL) using an Oxygraph-2k high-resolution respirometer (Oroboros Instruments, Innsbruck, Austria) at 37 °C with continuous stirring. We equilibrated 2 mL of respiratory medium (MiR05) in each chamber at 37 °C, and oxygen concentration was continuously monitored throughout the experiment. Cells were permeabilized with saponin (125 µg/mL). Mitochondrial respiration was assessed using a substrate–uncoupler–inhibitor titration (SUIT)-type protocol. Glutamate (5 mmol/L) and malate (2 mmol/L) were first added to provide electron donors to complex I (CI), and oxygen consumption was recorded under non-phosphorylating (leak) conditions in the absence of ADP (CI-linked LEAK). ADP (2 mmol/L) was subsequently added to stimulate ATP synthase and determine CI-linked oxidative phosphorylation (OXPHOS CI). Succinate (25 mmol/L) was then added to stimulate complex II (CII), allowing assessment of combined CI- and CII-linked oxidative phosphorylation (OXPHOS CI + CII). Rotenone (0.5 µmol/L) was subsequently added to inhibit CI and determine CII-linked oxidative phosphorylation (OXPHOS CII). Finally, TMPD and ascorbate (0.5 mmol/L each) were added to assess complex IV (CIV)-linked respiratory capacity by providing electrons directly to CIV. Oxygen flux was continuously recorded and expressed as pmol O_2_/s/million cells.

### 2.3. Statistical Analysis

A power analysis was performed using G*Power (version 3.1.9.7, Heinrich-Heine-University, Düsseldorf, Deutschland) to determine the required sample size, assuming a mitochondrial respiration difference of 20% and a statistical power of 80% and an alpha level of 5%. The number of patients required was 10 per group.

Statistical analyses were carried out using GraphPad Prism software (version 8.4.3, Boston, MA, USA). Quantitative data were expressed as mean ± standard error of the mean (SEM). The normality of the value distribution was analyzed according to a Shapiro–Wilk test. Patient and control characteristics were compared according to a non-parametric Mann–Whitney test used to compare the control and the AS groups. To compare several parameters with one another, we used the Kruskal–Wallis test followed by Dunn’s test. The observed correlations were analyzed with a non-parametric Spearman’s test. When we compared the effects at Days 0, 3, and 30 in the same patient, we used a Friedmann test. The *p*-value has been set at 0.05.

Moreover, we conducted multivariate analysis investigating the relative role of gender, age, and trans-aortic valve gradient in RCR and complex IV variations and of complex IV, age and trans-aortic valve gradient in AS patients’ mortality. This was performed using JMP software, version 7.0 (SAS Institute Inc., Cary, NC, USA).

## 3. Results

### 3.1. Clinical Characteristics of the Population

The main clinical characteristics of the controls and AS patients are presented below. Age and gender were not similar in the two groups (Table 1) and unlike healthy controls, patients had medical history such as systemic hypertension, dyslipidemia, and/or diabetes.

### 3.2. Biological Characteristics of the Patients

In Table 2, the main biological characteristics of AS patients are presented and compared to the normal laboratory range.

Overall, AS patients had normal or near-normal laboratory values. However, BNP levels were elevated compared to normal values. The cell count was normal. Markers of inflammation were present in the AS group, as inferred by increased neutrophil- and leukocyte-to-lymphocyte ratio.

### 3.3. Echocardiographic Characteristics of the Patients

Table 3 shows the main left and right heart echocardiographic characteristics of the patients. Left and right heart contractility and cardiac index were in the normal ranges. As expected in AS, the trans-aortic valvular gradient was increased, leading to left heart hypertrophy. The increased mitral E/E’ ratio correspond to an increased left atrial pressure linked to cardiac diastolic dysfunction.

### 3.4. Decreased Mitochondrial Coupling and Increased Complex IV Mitochondrial Respiration in the PBMC of AS Patients

Coupling is defined by the ratio of OXPHOS CI to CI leak. In the AS group, the RCR is significantly lower in AS patients than in the control group (2.12 ± 0.10 vs. 2.76 ± 0.17, *p* = 0.003), as shown in Figure 1.

There is no difference between the control group and the AS group in the OXPHOS analysis, regardless of the complex. However, the activity of complex IV is significantly increased in the AS group compared to the control group (31.07 ± 2.84 vs. 18.69 ± 2.09 pmol/sec/million cells, *p* = 0.017).

To go further in mitochondrial changes explanations, we assessed more specifically gender and age since the control were not perfectly matched to the patients, particularly considering these parameters.

Considering gender, we compared mitochondrial coupling and complex IV-related respiration in men and women both in controls and in AS patients (Figure 2). As shown below, the mitochondrial values were similar whatever the gender in both groups.

Concerning age, the AS patients were older than the healthy controls. To discriminate the potential role of age, in addition to that of AS, in mitochondrial changes, we present the data with a cut-off value of below or more than 72 years, which is the mean age of the control population (Figure 3). Mitochondrial coupling is similar whatever the age group in both controls and AS patients. Further, complex IV respiration was not significantly different between subgroups.

Finally, to complete these results, we conducted a multivariate analysis and a positive, but non-significant trend was observed between complex IV and age (r = 0.36; *p* = 0.08).

Considering therapy and comorbidities, mitochondrial respiration was similar in patients taking or not statins and treated or not for diabetes or systemic hypertension.

Finally, to investigate whether mitochondrial abnormalities predict adverse prognosis, we determined mortality as a long-term clinical endpoint. Interestingly, mortality was lower in AS patients presenting with a greater complex IV respiration (Figure 4a). Further, multivariate analysis showed that complex IV alone provided good discrimination (AUC = 0.825). Adding age and aortic transvalvular gradient yields only a marginal gain.

### 3.5. No Change in PBMC Mitochondrial Coupling and Respiration Post-TAVR

To determine whether reduction in the transaortic gradient might be associated with a normalization of mitochondrial respiration, we analyzed mitochondrial respiration and coupling three and thirty days after TAVR. The data were obtained in all the 25 patients at the three points, J0, J3 and J30, and we observed no change in mitochondrial respiration over time (Figure 5).

## 4. Discussion

The main results of this study are that patients with severe aortic stenosis demonstrated impaired PBMC mitochondrial coupling together with increased complex IV mitochondrial respiration. Such mitochondrial changes were not statistically linked to gender, age or patients’ biological characteristics nor with AS consequences on heart systolic and diastolic functions. Nevertheless, increased complex IV mitochondrial respiration was associated with reduced mortality in AS patients. Finally, the TAVR failed to normalize mitochondrial coupling and complex IV respiration until 30 days after the procedure.

The main role of the mitochondrial respiratory chain is to generate ATP, the principal source of energy for the cells. This is particularly important in the heart, characterized by a high oxidative capacity, allowing all life-long energy-dependent systolic and diastolic activities. Cardiomyocyte mitochondrial impairments are involved in heart diseases pathophysiology, including in dilated, ischemic and valvular cardiomyopathy [24]. Particularly, impaired mitochondrial respiration has been observed after pressure overload such as after aortic stenosis or systemic hypertension. Thus, mild hypertrophy impaired mitochondrial oxidative capacity and reduced transmural complex IV activity [25,26]. Further, when systemic hypertension and/or aortic stenosis-induced left ventricular hypertrophy became maladaptive, leading to heart failure, ventricular mitochondrial dysfunction likely participated in patient’s prognosis. Accordingly, enhanced post-operative LV mass regression after aortic valve replacement for AS was associated with improved long-term survival [27].

To further investigate the involvement of mitochondria in the setting of AS, rather than cardiac biopsy, PBMC mitochondrial respiration—simply needing blood withdrawal—might be particularly interesting since it has been proposed to reflect cardiac muscle alterations. Indeed, studies suggest that PBMC may be a non-invasive novel biomarker of heart failure and surrogate for myocardial mitochondrial respiratory function. In heart failure patients, inflammation and severity of the disease were associated with PBMC mitochondrial dysfunction [18,19,20,21,28].

We therefore investigated the mechanisms potentially involved in the mitochondrial respiration changes observed in our cohort of AS patients, analyzing their clinical, biological and echocardiographic characteristics.

### 4.1. Aortic Stenosis Is Associated with Impaired Mitochondrial Coupling

Globally, the PBMC mitochondrial respiration of AS patients is similar to that of controls. Thus, the oxidative capacity through complex I and II is maintained. Nevertheless, mitochondrial coupling between oxidation and phosphorylation, represented by the respiratory control ratio (RCR), is decreased in AS patients. Li. et al. [19] described a global decrease in mitochondrial respiratory function in early-stage, asymptomatic heart failure with preserved ejection fraction as compared to controls. As in our study, these patients presented cardiac remodeling (cardiac hypertrophy and/or a delayed relaxation), cardiometabolic risk factor (systemic hypertension, dyslipidemia and diabetes) and increased markers of inflammation.

The mechanisms potentially involved in PBMC-reduced mitochondrial coupling during AS are unknown, but we investigated whether similar parameters might explain our results.

Age and gender deserve discussion since our control group had more men than women and was younger than the AS group. Indeed, it was difficult to find very old healthy people who could mirror the very old patients needing TAVR well. Such a limitation is also found in the literature where reports concerning PBMC mitochondrial function in subjects around 80 years old are scarce. Controversial data have been reported, but globally it seems that, without comorbidities, age alone has relatively little influence on PBMC mitochondrial respiration. Indeed, although increased age was associated with increased maximal respiration, this pattern varied with gender (mainly true in women) and probably more generally with the context of the study [29,30]. Thus, if any role, older age might solely be a cofactor in this context.

Concerning gender, Fliegner et al. investigated potential sex differences in cardiac mitochondrial respiration in humans with AS. The authors reported higher mitochondrial State-3 respiration in female than that in male human heart biopsies, with experimental data confirming a link with estrogen levels [31]. We did not observe such a relationship in our study in PBMC, with gender being not associated with differences in mitochondrial respiration in neither controls nor AS patients.

Cardiovascular metabolic risk factors, including systemic hypertension, dyslipemia and diabetes, largely associated with cardiovascular and renal diseases development and chronic inflammation might participate in the impaired mitochondria observed in AS patients [32,33,34,35,36]. Further, neutrophils are known to facilitate cardiomyocytes’ apoptosis [37] and relative lymphopenia might occur due to the inflammation and down-regulation of the immune system [38]. However, we found nonsignificant relationship between these parameters and the mitochondrial coupling in the AS patients.

### 4.2. Aortic Stenosis Is Associated with Increased Complex IV Mitochondrial Respiration

The complex IV is the final enzyme of the electron transport chain system in mitochondria, importantly modulating the oxidative phosphorylation [39,40]. Increased complex IV activity might be viewed as a compensatory mechanism for the decreased mitochondrial coupling respiration observed in AS patients.

To go further, considering the inverse correlation previously reported between isovolumic relaxation time and the activity of complex IV in heart transplant recipients [22], we expected that cardiac diastolic function and parameters linked to pressure overload be associated with the increased complex IV activity in our AS patients. This was not the case, whatever the echocardiographic parameters analyzed.

We then investigated the role of statin therapy. Indeed, statin treatment (generally associated with decreased inflammatory markers) can increase complex IV mitochondrial respiration in PBMC compared to untreated controls [41]. However, the mitochondrial characteristics were similar in our AS patients, whether they were taking statins or not, eliminating such a possibility. Mitochondrial respiration was also similar in patients, regardless of whether they were being treated for diabetes or systemic hypertension.

Interestingly, mortality was reduced in AS patients presenting with an increased complex IV mitochondrial respiration, supporting its prognosis value and confirming a compensatory role of stimulated complex IV activity in cardiovascular diseases [22,40].

### 4.3. Hemodynamically Successful TAVR Did Not Normalize Mitochondrial Respiration

Successful TAVR allows an almost immediate near normalization of the trans-aortic valve gradient, waiving pressure overload. This was shown to be associated with a rapid decrease in circulating oxidative stress [23]. In our study, even 30 days after the TAVR procedure, no change was observed in mitochondrial coupling or complex IV respiration, but 30 days may be too short to determine whether mitochondrial remodeling truly “persists” after correction of the valvular lesion since reverse ventricular remodeling and systemic inflammatory/metabolic changes may require substantially longer periods.

#### Limitations of the Study

This study had several limitations that can limit its generalizability. Although allowing significant changes in PBMC mitochondrial respiration to be observed, this single-center work included a relatively small number of 25 AS patients with a follow-up 30 days after TAVR. Unfortunately, it was difficult to find well-matched controls demonstrating a single abnormal parameter characterizing the AS patients. Although multivariate analysis allowed discrimination between some parameters, further larger studies are needed before generalizing these data. Correction for multiple testing was not applied considering biological and echocardiographic variables, the number of which might have been below 25 since collection on medical charts was retrospective. We assumed that PBMC mitochondrial respiration should not be different whether taken by femoral or antecubital venipuncture. Indeed, in both cases, this corresponds to peripheral venous blood and it is assumed generally that differences in biological parameters occur mainly when comparing venous and arterial sampling. Further, very significant modifications are needed to acutely change PBMC metabolism, like, for instance, during severe hypoxia [42]. Nevertheless, to the best of our knowledge, there is no data demonstrating comparability between the two sampling approaches and this is a limitation of our study.

## 5. Conclusions

This study shows, for the first time in patients with severe aortic stenosis, abnormal PBMC mitochondrial function, associated with reduced coupling and increased complex IV mitochondrial respiration. Severe aortic stenosis is related to bioenergetic remodeling of circulating mononuclear cells, but is not significantly associated with valvular hemodynamic impairment and ventricular function, nor is it corrected 30 days after the mechanical relief of afterload.

Nevertheless, increased complex IV mitochondrial respiration is associated with reduced mortality supporting larger studies to determine whether such interesting systemic immuno-metabolic phenotype might be a clinically useful biomarker contributing to approach the residual risk following TAVR. Indeed, PBMCs are continuously exposed to the inflammatory and hemodynamically stressed environment generated by the stenotic valve and remodeled myocardium. Therefore, alterations in their mitochondrial bioenergetic profile might reflect both systemic and cardiac disease progression, might disappear after hemodynamic and cardiac normalization, and may potentially be modulated to improve cardiovascular functions [43].

## Figures and Tables

**Figure 1:**
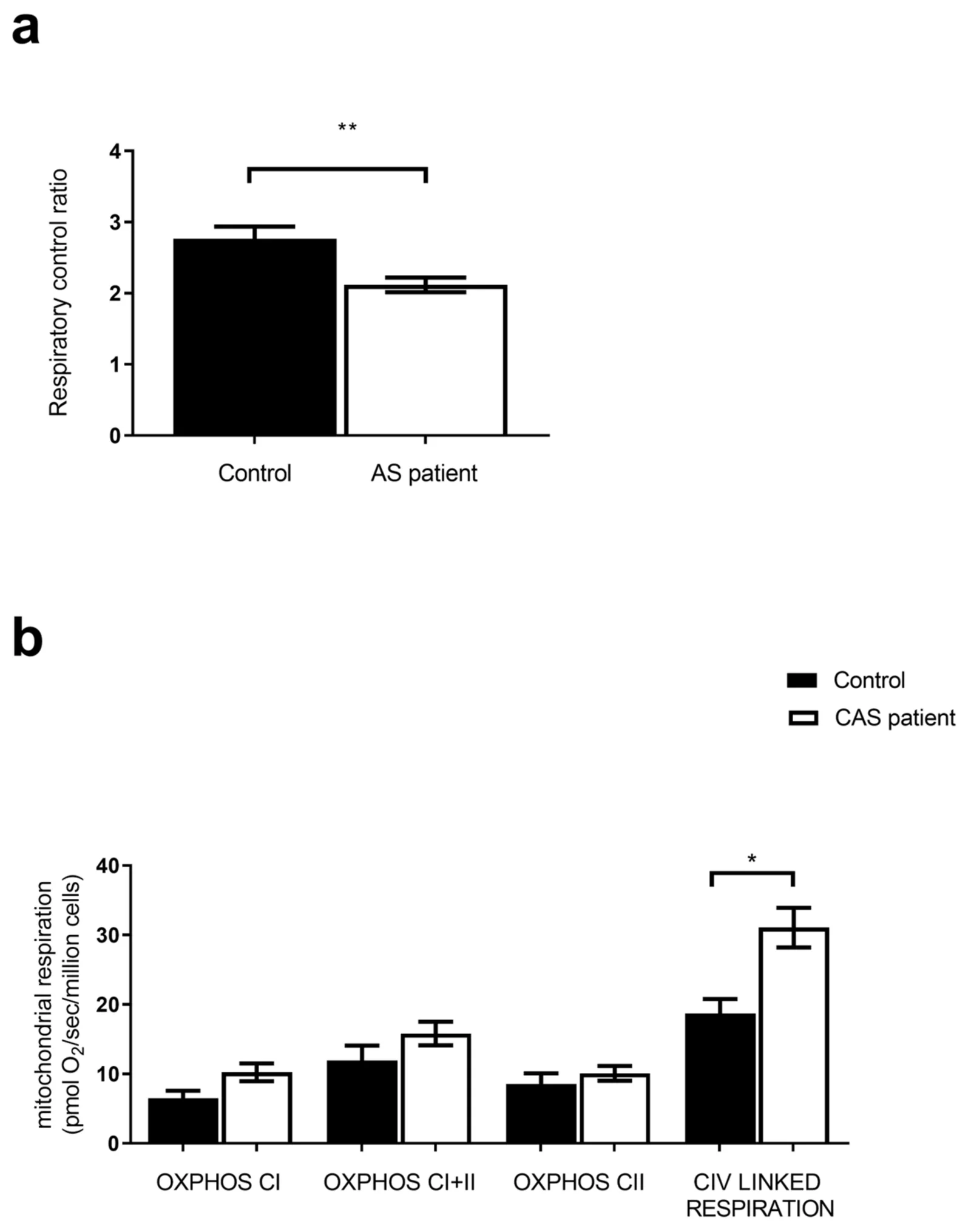
Reduced mitochondrial coupling and increased complex IV respiration in AS patients compared to controls. (**a**) Mitochondrial coupling is observed with RCR on isolated PBMC. (**b**) Mitochondrial respiratory capacity of OXPHOS CI, OXPHOS CI+CII, OXPHOS CII on PBMC. RCR: respiratory control ratio; OXPHOS: oxidative phosphorylation; CI: mitochondrial complex I; CI + II: mitochondrial complexes I and II; CII: mitochondrial complex II. CIV: mitochondrial complex IV. OXPHOS: mitochondria ADP-activated state of oxidative phosphorylation. AS: calcified aortic stenosis; values are means ± SEM, (*n* = 9 for the control group, and 25 for AS group). The statistical test used is an unpaired *t*-test for values that follow a normal distribution and a Mann–Whitney test for the others, *: *p* < 0.05, **: *p* < 0.01.

**Figure 2:**
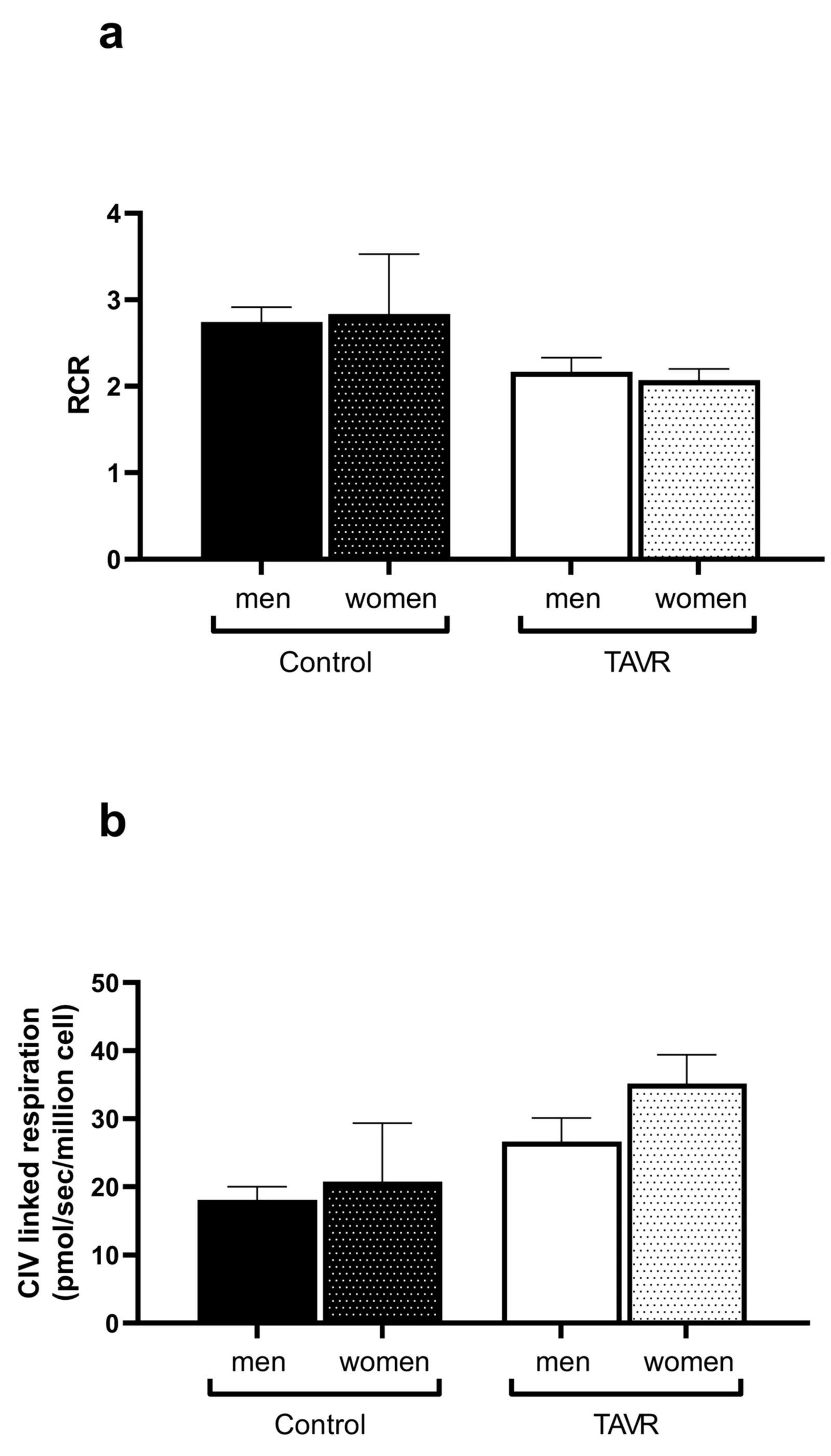
Mitochondrial coupling (**a**) and complex IV-related respiration (**b**) are similar in men and women controls and aortic stenosis patients. Values are means ± SEM.In the control group, *n* = 2 for women and *n* = 7 for men. In the TAVR group, *n* = 13 for women and *n* = 12 for men. The statistical test used is the Kruskal–Wallis test.

**Figure 3:**
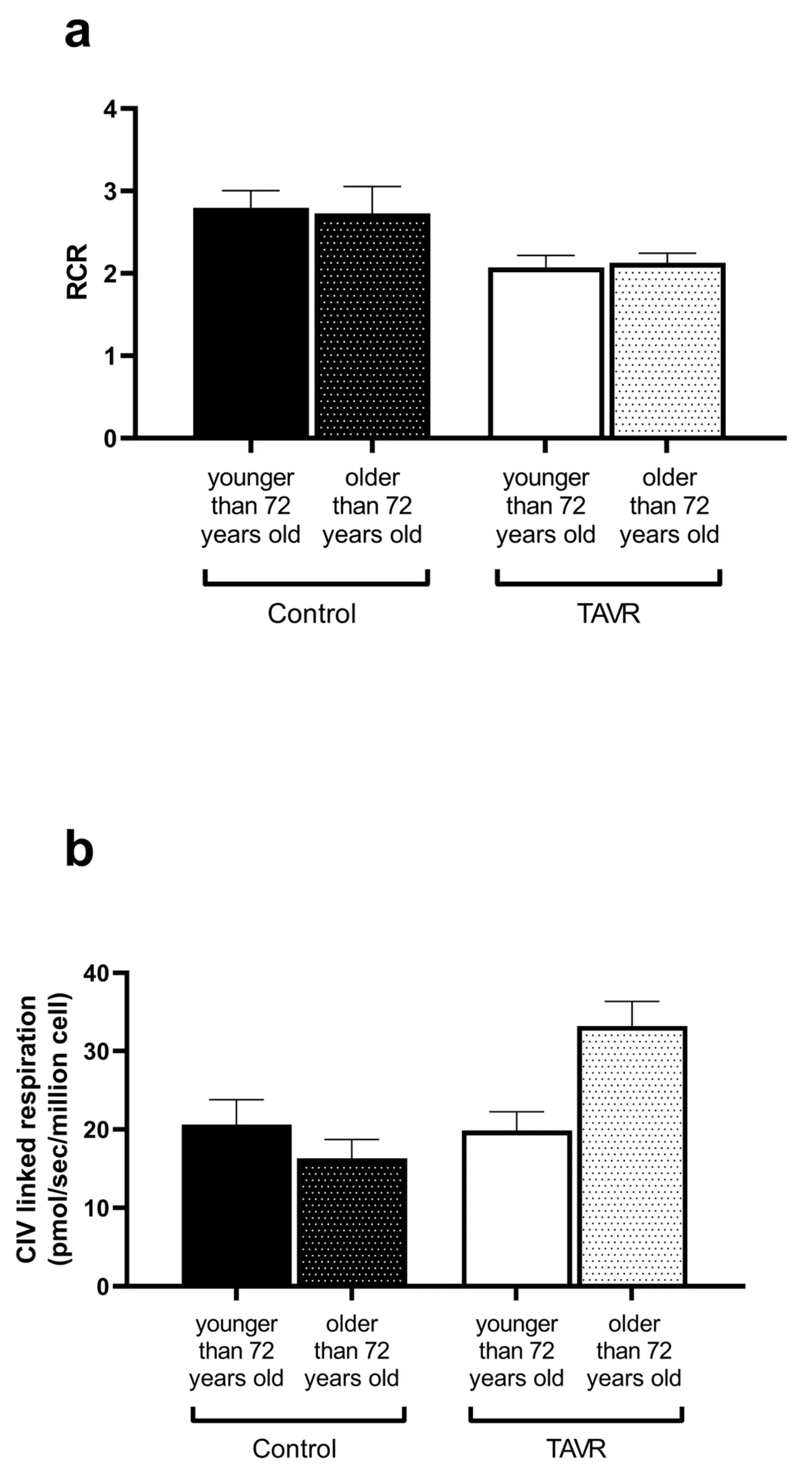
Mitochondrial coupling (**a**) and complex IV-related respiration (**b**) in controls and aortic stenosis patients younger and older than 72 years. In the control group, *n* = 5 for individuals under the age of 72 and *n* = 4 for individuals over the age of 72. In the TAVR group, *n* = 4 for individuals under the age of 72 and *n* = 21 for individuals over the age of 72. The statistical test used is the Kruskal–Wallis test.

**Figure 4:**
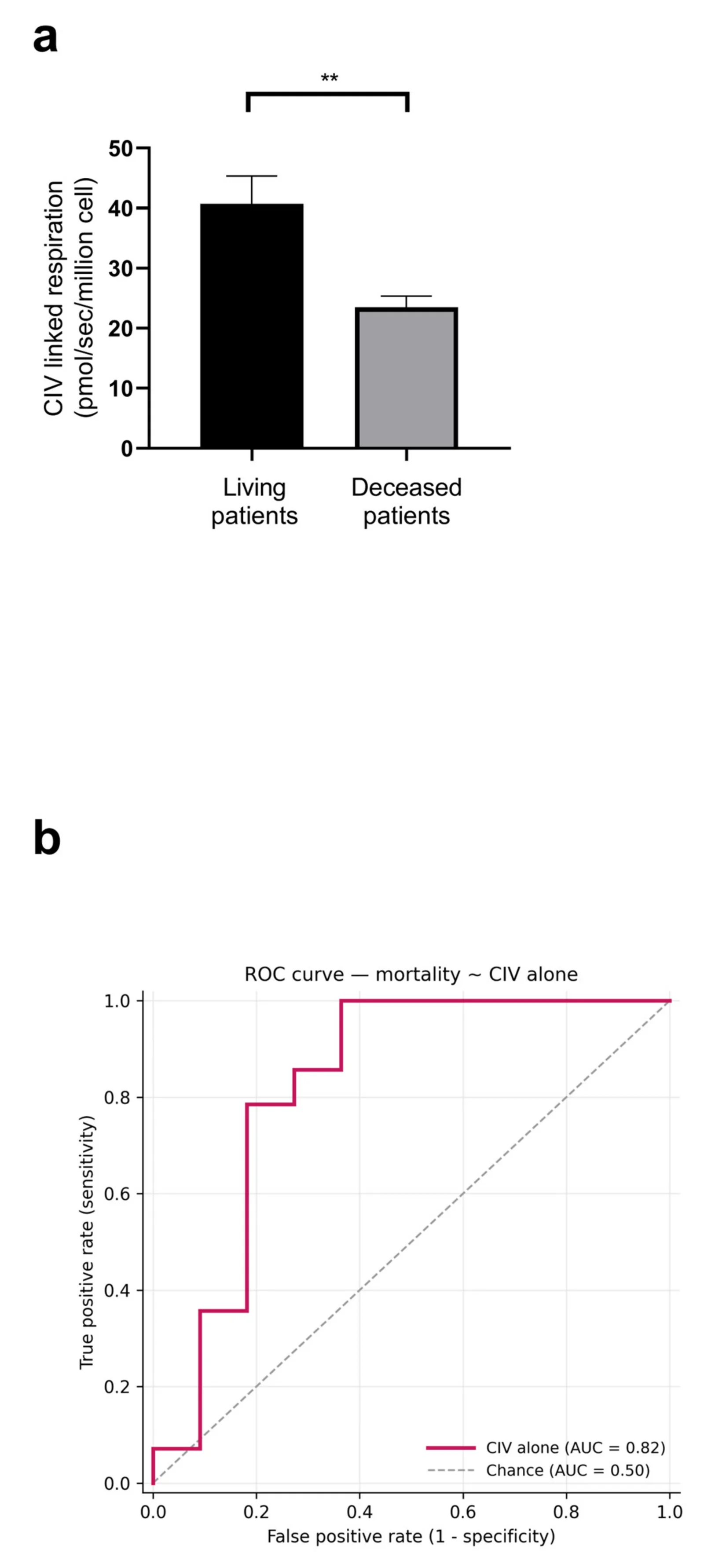
(**a**) Reduced mortality rate in AS patients is associated with increased complex IV mitochondrial respiration. Mitochondrial complex IV-related respiration in AS patients is a function of postoperative mortality. The statistical test used is the Mann–Whitney test, *n* = 11 for the subgroup of living patients, and *n* = 14 for the subgroup of deceased patients. **, *p* < 0.01. (**b**) The ROC curve analysis of complex IV-linked respiration for mortality prediction. The ROC curve illustrates the ability of CIV-linked respiration to discriminate between alive and deceased patients, *n* = 25. Values are means ± SEM.

**Figure 5:**
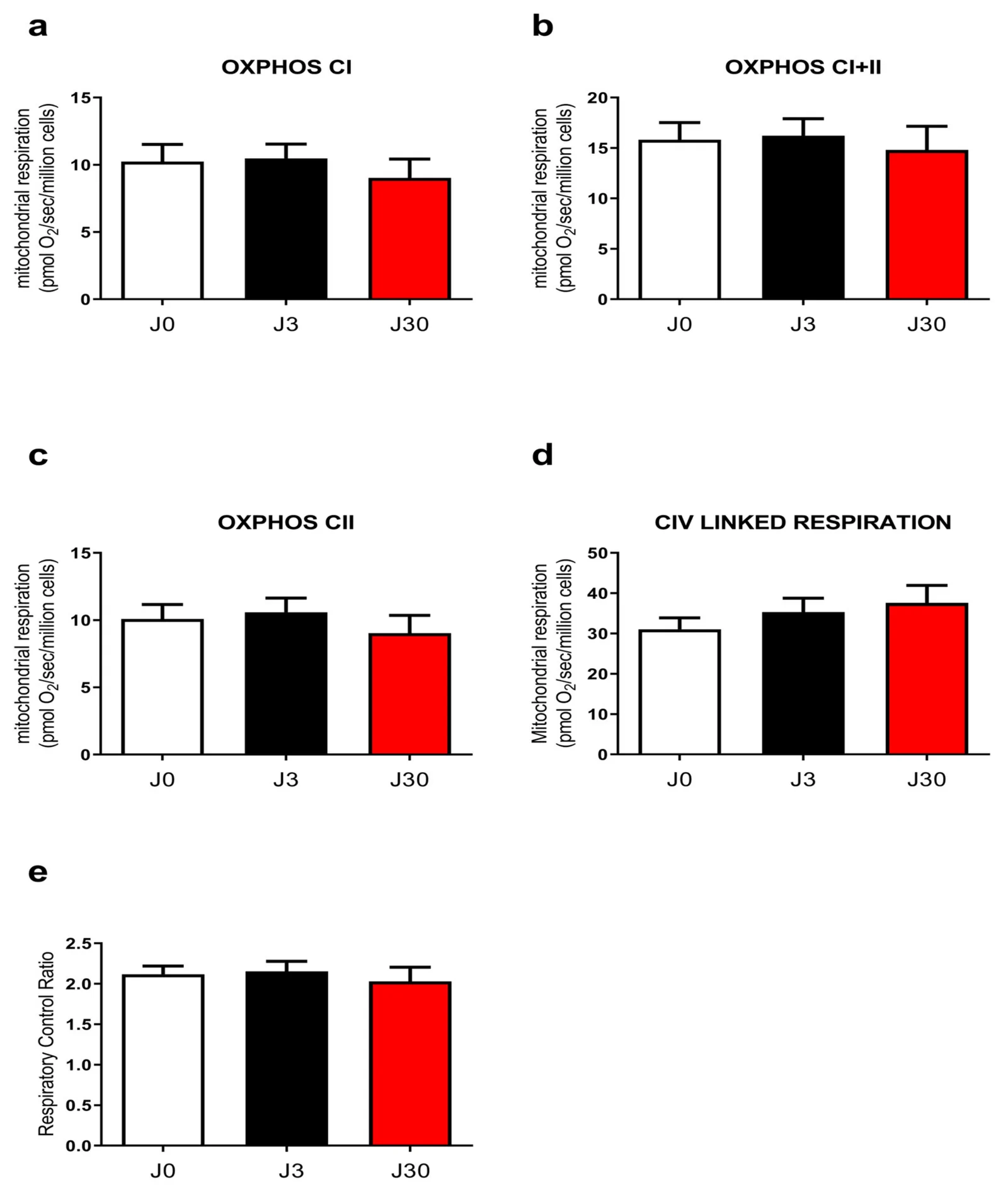
Kinetic of PBMC mitochondrial respiration in AS patients. Mitochondrial respiratory capacity of (**a**) OXPHOS by CI, (**b**) OXPHOS by CI+CII, (**c**) OXPHOS by CII, (**d**) CIV activity on isolated PBMC and (**e**) coupling. JO AS, J3 and J30 after TAVR. All values are expressed as the mean ± SEM. *n* = 25 at Jo, J3 and J30. A Friedmann test was used.

**Table 1:** Clinical characteristics of the population.

	Healthy Controls	AS Patients	*p* Value
Gender (M/F)	7/2	12/13	NS
Age (years)	72.33 ± 2.24	81.20± 1.64	*p* = 0.007
**Comorbidity (*n*)**			
Systemic hypertension	0	20	
Dyslipidaemia	0	17	
Diabetes	0	9	

AS: aortic stenosis. Results are expressed as mean ± SEM and compared to normal value range. NS: not significant.

**Table 2:** Biological characteristics of AS patients.

	Normal Values (Range)	AS Patients (Mean ± SEM)
Glycemia (g/L)	0.70–1.10	1.11 ± 0.11 (*n* = 19)
Creatinine (umol/L)	64–104	98.72 ± 7.13, *n* = 24
Glomerular filtration rate (mL/min/1.73 m^2^)	>90	59.67 ± 3.76, *n* = 24
Troponine (mg/mL)	<0.04	0.03 ± 0.008, *n* = 24
BNP (pg/mL)	10–150	410.1 ± 78.69, *n* = 24
Hb (hemoglobin level (g/dL)	12–16	12.34 ± 0.41, *n* = 24
**Blood Cells Count (×10^9^/L)**		
Leucocytes	4–11	6.99± 0.57, *n* = 24
Lymphocytes	1–4	1.38 ± 0.18, *n* = 16
Neutrophils	1.40–7.70	4.89 ± 0.55, *n* = 17
**Inflammation**		
CRP (mg/L)	<5.0	6.23 ± 2.38, *n* = 16
NLR (Neutrophil-to-lymphocyte ratio)	0.78–3.53	3.87 ± 0.37, *n* = 16
LLR (Leukocyte-to-lymphocyte ratio)	2.3–4	5.55 ± 0.45, *n* = 16

AS: aortic stenosis; BNP: brain natriuretic peptide; CRP: c-reactive protein. Results are expressed as mean ± SEM and compared to normal value range (descriptive comparisons). Neutrophil-to-lymphocyte ratio (NLR), and leukocyte-to-lymphocyte ratio (LLR).

**Table 3:** Echocardiographic characteristics of TAVR patients.

	Normal Values	AS Patients
Left ventricular ejection fraction (%)	>55	60.72 ± 2.08, *n* = 25
Right ventricular fractional shortening (%)	>32	44.52 ± 2.17, *n* = 20
Cardiac index (L/min/m^2^)	2.5–3.5	3.73 ± 0.37, *n* = 18
Systolic pulmonary artery pressures (PAPs) (mmHg)	<40	42.85 ± 2.69, *n* = 20
Mitral E/A ratio	1–2	0.96 ± 0.14, *n* = 18
E/E’ ratio	<8	13.94 ± 1.13, *n* = 18
Left ventricle mass (g/m^2^)	95–115	127.8 ± 7.36, *n* = 22
Trans-aortic valve gradient (mm Hg)	<10	45.0 ± 2.36, *n* = 25

E/A: mitral E and A wave’s ratio. E’: mean speed of lateral and medial wall using Doppler tissue imaging. Results are expressed as mean ± SEM and compared to normal value range (descriptive comparisons).

## Data Availability

The raw data supporting the conclusions of this article will be made available by the authors on request.
